# Safety and Efficacy of Cone-Beam Computed Tomography-Guided Lung Tumor Localization with a Near-Infrared Marker: A Retrospective Study of 175 Patients

**DOI:** 10.3390/life12040494

**Published:** 2022-03-28

**Authors:** Chia-Jung Chang, Chi-Hsuan Lu, Xing Gao, Hsin-Yueh Fang, Yin-Kai Chao

**Affiliations:** Division of Thoracic Surgery, Chang Gung Memorial Hospital-Linkou, Chang Gung University, Taoyuan 333423, Taiwan; chang0216@cgmh.org.tw (C.-J.C.); eric880323@cgmh.org.tw (C.-H.L.); xgao@cgmh.org.tw (X.G.); b9302067@cgmh.org.tw (H.-Y.F.)

**Keywords:** near-infrared marking, small pulmonary nodules, indocyanine green, hybrid operating room

## Abstract

Preoperative localization holds promise for overcoming the limitations of video-assisted thoracoscopic surgery (VATS) in the treatment of impalpable lung nodules. The purpose of this study was to assess the safety and efficacy of cone-beam computed tomography (CBCT)-guided localization using near-infrared (NIR) marking. Between 2017 and 2021, patients presenting with a solitary pulmonary nodule (SPN) who had undergone CBCT-guided lesion localization with indocyanine green (ICG) in a hybrid operating room were included. The primary outcomes were the efficacy of localization and the occurrence of complications. The study cohort consisted of 175 patients with the mean age of 58.76 years. The mean size and depth of the 175 SPNs were 8.34 mm and 5.3 mm, respectively. The mean time required for lesion marking was 14.71 min. Upon thoracoscopic inspection, the NIR tattoo was detected in the vast majority of the study participants (98.3%). An utility thoracotomy to allow digital palpation was required in two of the three patients in whom the tattoo was not identifiable. The perioperative survival rate was 100%, and the mean length of hospital stay was 3.09 days. We conclude that needle localization with ICG injection is a safe and feasible technique to localize SPNs prior to resection.

## 1. Introduction

Recent years have witnessed an increased volume of radiological investigations applied to lung cancer screening [1,2]. As a result, the number of video-assisted thoracoscopic surgery (VATS) procedures aimed at removing small-sized single pulmonary nodules (SPNs) through wedge resection climbed sharply. Unfortunately, instrumental palpation of the lung parenchyma may fail to localize small and/or deeply located target lesions [3]. Inadequate tumor localization during surgery is associated with several shortcomings—including a prolonged operating time, an increased risk of conversion to thoracotomy, and, in a worst case scenario, the failure to remove the target nodule [4]. Consequently, several preoperative marking techniques have been developed with the goal of improving lesion localization and optimizing subsequent removal [5].

While rapid technical advances are furthering the application of these procedures, their implementation in clinical practice is not without caveats. Hookwire localization has been repeatedly shown to be effective [6], but the risk of dislodgment remains a major hurdle [5,7]. As potential alternatives, the use of microcoils/fiducials or water-insoluble contrast media (e.g., barium or lipiodol) has been proposed [8,9,10,11,12] although intraoperative radiation exposure remains far from negligible. Another technique based on the use of patent blue V (PBV) or methylene blue dye may successfully localize small, superficial tumors [13,14,15]; however, it is of limited utility for deeply located (>1 cm from the pleural surface) lesions owing to the suboptimal penetration depth of the dye into the lung parenchyma [16].

By taking advantage of a greater penetration compared with traditional dye markers and the ability to offer a radiation-independent localization approach, the use of fluorescent dye characterized by absorption and emission wavelengths in the low-energy near-infrared (NIR) spectrum may circumvent the issues inherent in other techniques [16]. To date, a total of four fluorescent dyes have been used in the field of general thoracic surgery—either in everyday practice or in the context of clinical trials [17,18,19]. They include indocyanine green (ICG) and the three receptor-targeted agents (EC17, OTL38, and 5-aminolevulinic acid (5-ALA)) [18]. Currently, the most commonly used NIR fluorescence dye is ICG (excitation and emission wavelengths: 778 nm and 830 nm, respectively)—which was granted approval from the Food and Drug Administration in 1959 [20]. Despite its longstanding use, published data on the clinical utility of ICG localization followed by NIR thoracoscopic resection of SPNs remain limited [21,22]. The present study was therefore designed to assess the safety and efficacy of ICG-guided lung tumor localization using a percutaneous cone-beam computed tomography (CBCT)-guided approach implemented in a hybrid operating room (HOR).

## 2. Materials and Methods

We retrospectively reviewed the clinical records of patients presenting with a solitary pulmonary nodule (SPN) who had undergone CBCT-guided lesion localization with ICG in a HOR between July 2017 and May 2021. The study complied with the tenets of the Helsinki Declaration and was granted ethical approval by the Institutional Review Board (reference: CGMH-IRB 201600671A3). The need for patient consent was waived due to the study design.

### 2.1. Indications for Preoperative Tumor Localization

According to our institutional policy, the criteria for lesion localization included the presence of (1) ground glass opacities (GGOs) or (2) subsolid or cavitary lesions. Additionally, we localized subpleural solid nodules < 10 mm in size as well as solid nodules deeply located in the lung parenchyma (distance from the visceral pleural surface ≥ 10 mm).

### 2.2. Localization Procedure and ICG Preparation

One vial of ICG (25 mg; Diagnogreen; Daiichi-Sankyo Co., Ltd., Tokyo, Japan) was initially dissolved in 10 mL of distilled water. Prior to the marking procedure, a 1 mL syringe and its needle lumen were prefilled with the ICG solution. The procedural workflow applied for HOR localization has been previously described in detail [23,24]. In brief, following the induction of general anesthesia, patients were placed in the lateral decubitus position. Both the CBCT C-arm and the patient’s chest were protected with sterile wraps. An initial scan for surgical planning was acquired during end-inspiration breath-hold using a standard 6 s DynaCT Body protocol. Under the syngo Needle Guidance provided by the syngo X-Workplace (Siemens Healthcare GmbH, Erlangen, Germany), the access path was laid out in the isotropic data. After outlining the needle path by marking the needle entry and target points, it was subsequently projected with a laser beam onto the patient’s skin. Following puncture of the lung parenchyma, the ICG solution (0.3 mL) was injected in the proximity of the nodule.

Upon VATS initiation, real-time intraoperative NIR fluorescence images were acquired using either a minimally invasive ICG fluorescence system (PINPOINT (Stryker, Kalamazoo, MI, USA)_or a D-Light (Karl Storz, Tuttlingen, Germany)) that included a 10 mm, 30-degree NIR thoracoscopic camera for identifying the NIR tattoo. VATS wedge resection was subsequently carried out using surgical staplers under NIR tattoo guidance. The resected pulmonary specimen underwent frozen section examination. Upon confirmation of primary lung cancer, a lobectomy was performed when the tumor size was >2 cm or in presence of inadequate surgical margins.

### 2.3. Definition of Outcomes

The duration of localization was defined as the time elapsed from patient positioning and C-arm docking to the end of localization. The time at risk was defined as the time interval between the completion of localization and skin incision. A procedure was considered technically successful when ICG was visible on the pleural surface during the course of VATS. Procedural complications were classified into two distinct categories (i.e., pneumothorax and lung hemorrhage), and their occurrence was investigated during the CT scan that immediately followed lesion localization. According to the 2010 British Thoracic Society guidelines, pneumothorax was defined as small when the distance from the lung margin to the chest wall was <2 cm; conversely, a large pneumothorax was considered to be present when the distance was ≥2 cm [25]. The Clavien–Dindo (CD) criteria were used to assess the severity of complications—with those classified as grade IIIa or higher being regarded as severe [26].

### 2.4. Statistical Analysis

The following variables were collected from clinical records: demographic data (age, sex, and smoking status), lesion characteristics (size, number, and location of nodules), information on the surgical approach (wedge resection, segmentectomy, or lobectomy), length of post-operative hospital stay, and data on in-hospital morbidity and mortality. Descriptive statistics were used to provide a summary of the study variables. Continuous variables are expressed as means ± standard deviations, whereas categorical data are given as counts and percentages. Analyses were performed using the Statistical Package for the Social Sciences (SPSS), version 20 (IBM, Armonk, NY, USA).

## 3. Results

The study cohort consisted of 175 patients (78 men and 97 women; mean age: 58.76 ± 10.92 years; Table 1). Of the 175 SPNs identified in our study, 101 and 74 were solid nodules and subsolid/pure GGOs, respectively. The mean size and depth of SPNs were 8.34 mm and 5.3 mm, respectively.

**Figure 1:**
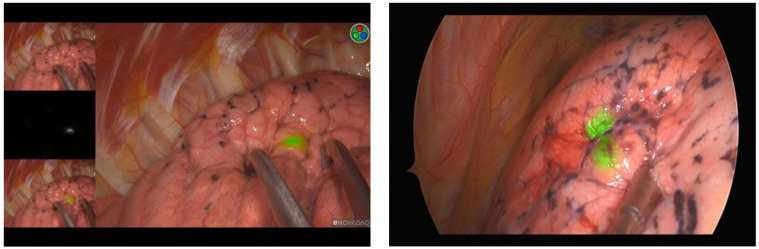
Intraoperative imaging using a NIR thoracoscope: evidence of minimal (**A**) or mild (**B**) fluorescence diffusion.

Table 2 summarizes the main localization and perioperative outcomes. The mean duration of localization was 14.71 ± 6.02 min. Six patients suffered from procedure-related small pneumothorax, but drainage was not required. No severe complications (e.g., cerebral air embolism or pulmonary hemorrhage) were observed. Upon thoracoscopic inspection, we were able to detect the NIR tattoo on the pleural surface in the vast majority of the study participants (98.3%, 172 out of 175; Figure 1). The mean time elapsed from the injection of ICG to skin incision (i.e., time at risk) was 13.67 ± 7.47 min. In one patient, there was an ICG spillage on the pleural surface that resulted in a diffuse illumination of the entire lung. Extravasation of ICG was easily be wiped off with a gauze, and surgery was carried out uneventfully.

**Figure 2:**
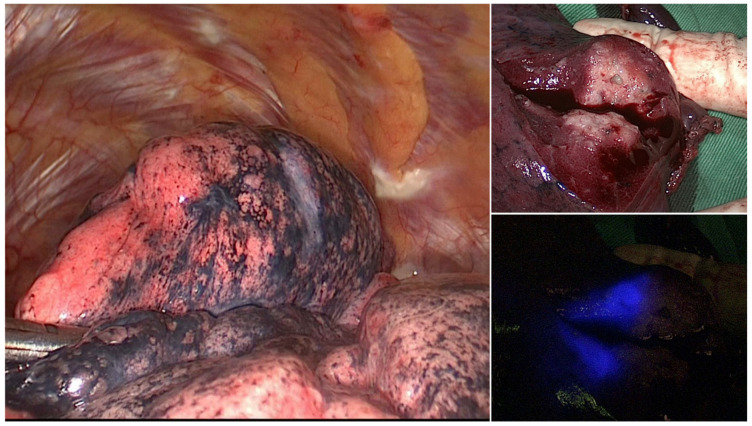
ICG localization failure: the gross inspection of the resected specimen (right upper panel) revealed an erroneous injection of the ICG dye into the deep lung parenchyma (right lower panel). This in turn resulted in the inability to visualize the NIR tattoo on the pleural surface (left panel).

An utility thoracotomy to allow digital palpation was required in two of the three patients in whom the tattoo was not identifiable. The lesions were subsequently removed through wedge resection under needle puncture guidance. The remaining case underwent blind segmentectomy. All of these three patients had tumors located within 10 mm from the pleural surface. Notably, gross inspection of the resected specimen revealed that the lack of visualization of the NIR tattoo on the pleural surface was attributable to an erroneous injection of the ICG dye into the deep lung parenchyma (Figure 2). As for the patients in whom the NIR tattoo was visible, SPNs were successfully removed through wedge resection in all cases. The results of pathology revealed that most SPNs (63.4%) were either primary lung cancer or lung metastases. Six patients with primary lung cancer received segmentectomy following a wedge biopsy that revealed an inadequate margin distance.

The mean duration of chest tube drainage was 1.63 ± 1.68 days. Two patients required prolonged (>5 days) drainage either because of persistent air leakage (*n* = 1) or empyema (*n* = 1). None of them required repeated surgery. The mean length of stay was 3.09 ± 2.14 days, and all of the study patients were successfully discharged home. The in-hospital and 90-day survival rates were both 100%.

## 4. Discussion

As of its introduction in our hospital in 2007, CT-guided percutaneous lung tumor localization has been mainly accomplished using a hookwire marker [27]. Unfortunately, this procedure has been limited by the occurrence of wire dislodgement in up to 7% of all cases [27]. An alternative approach to lesion localization lies in the use of PBV or methylene blue dye [13,14]. However, their limited tissue penetration represents a significant shortcoming in presence of deeply located pulmonary lesions (i.e., those with a distance > 10 mm from the pleural surface). In the current study, we described the procedural and surgical outcomes of 175 patients who had undergone ICG localization of SPNs. On analyzing lesions located within 20 mm from the pleural surface, we found that this approach allowed a reliable detection of the NIR tattoo in the vast majority of the study participants (98.3%). Our findings are in accordance with those of a previous animal study showing that the ICG signal was detectable at a maximum depth of 24 mm from the inflated lung surface [28]. Collectively, these results indicate that ICG marking of SPNs holds great promise to overcome the current technical shortcomings inherent in lung tumor localization, ultimately offering an effective solution to be applied before routine thoracoscopic surgery.

**Figure 3:**
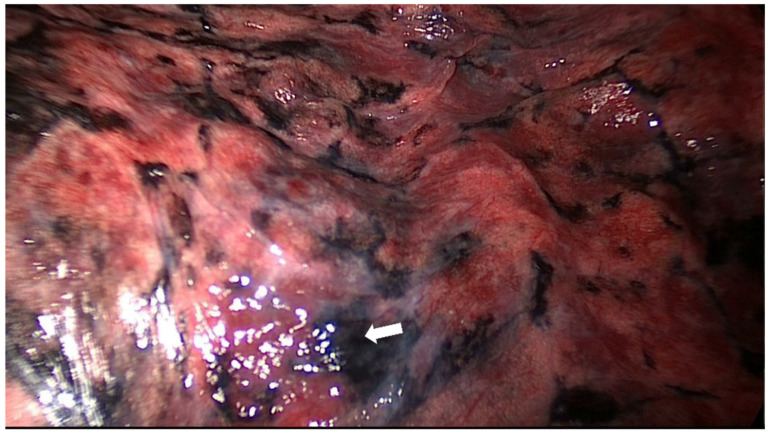
The identification of PBV dye (white arrow) under a white-light endoscope can be technically challenging in presence of color or texture changes of the visceral pleura.

Another advantage of ICG compared with the PVB or methylene blue dye is the possibility to correctly localize SPNs even in patients with anthracosis or other pulmonary diseases associated with color and/or textural changes of the visceral pleura [15]. While the identification of PBV or methylene blue dye under a white-light endoscope can be technically challenging in presence of color or texture changes of the visceral pleura (Figure 3), the specific wavelength of ICG fluorescence allows circumventing these issues.

Despite these promising results, the optimal parameters related to ICG injection have not yet been entirely elucidated and deserve further comment. Published studies focusing on pulmonary lesions differed significantly both in terms of ICG volume (0.1–1 mL) and concentration (0.125–12.5 mg/mL; Table 3). While a higher ICG concentration may theoretically increase the ability to visualize the NIR tattoo, it can also interfere with the results of pathological examination. Another potential shortcoming inherent in the use of NIR marking is shared with other dye markers. Specifically, the accuracy of target area identification is markedly affected by the time elapsed from tumor localization and subsequent thoracoscopy. In this scenario, surgery should be performed as rapidly as possible (i.e., within 3 h) to avoid the diffusion of ICG into the surrounding lung—which would lead to the inability to localize the lesion of interest [25]. One of the strengths of our study was that all procedures were performed in a HOR—which allowed us to minimize the time elapsed from localization to surgery [29]. Further research is needed to establish the optimal safety window between ICG localization and subsequent surgery.

In addition to percutaneous injection in the peritumoral area, systemic administration of ICG has emerged as a useful strategy to facilitate the identification of lung malignancies—albeit through a completely different mechanism. In this regard, numerous studies have shown that ICG given intravenously (dose: 1–5 mg/kg) within 24 h before surgery allowed detecting 90–91% of all pulmonary nodules [33,34,35]. While this occurs through an enhanced permeability and retention effect, ICG cannot be considered as a tumor-specific fluorescence dye. Accordingly, accumulation occurs solely in certain tumor types (e.g., pulmonary metastases from hepatocellular carcinoma and hepatoblastoma) [36]. The question as to whether the systemic administration of ICG can facilitate localization of early-stage lung cancers presenting as GGOs or subsolid lesions remains open. Another limitation pertains to the risk of ICG diffusion in areas of vascular hyperpermeability (e.g., in case of inflammation or ischemic injuries). Based on this evidence as a whole, we believe that systemic ICG may be sensitive but not specific in identifying SPNs. Additional optimization of NIR targeting agents will be required to improve their positive predictive values.

Several caveats of our study must be considered. First, all of the ICG localizations implemented in our research were performed on tumors with a depth of less than 2 cm. The question as to whether this approach could be safely applied to deeply located lung tumors remains unanswered. Second, the equipment required for ICG-based thoracoscopic visualization of SPNs may find high-cost barriers. Third, the single-center retrospective design of our study may have limited the external validity of the results, and for that reason, larger prospective cohorts are needed to confirm the safety and efficacy of CBCT-guided ICG dye localization. More studies are also necessary to compare this new technique with previously established localization approaches by taking into account the diagnostic yields, the complication rates, and cost effectiveness as the main outcomes of interest [37,38,39].

## 5. Conclusions

In summary, the results of our study indicate that the ICG dye allows a safe and reliable localization of SPNs—which represents a crucial prerequisite to successful VATS removal. With the widespread implementation of lung cancer screening programs, our findings may have significant diagnostic and therapeutic implications.

## Figures and Tables

**Table 1:** General characteristics of the study participants (*n* = 175).

Variable	Value
Age, years	58.76 ± 10.92
Sex, *n* (%)	
Man	78 (44.6%)
Woman	97 (55.4%)
Tumor size, mm	8.34 ± 3.64
Tumor depth, mm	5.30 ± 4.53
Tumor location, *n* (%)	
Right	105 (60%)
Left	70 (40%)
Nodule characteristics, *n* (%)	
Solid	101 (57.7%)
Partially solid or GGO	74 (42.3%)

Continuous variables are expressed as means ± standard deviations, whereas categorical data are given as counts and percentages. Abbreviation: GGO, ground-glass opacity.

**Table 2:** Procedural and surgical variables in the study participants (*n* = 175).

Variable	Value
Mean time required for localization, min	14.71 ± 6.02
Time at risk, min	13.67 ± 7.47
Procedural complications, *n* (%)	
Pneumothorax	6 (3.4%)
Hemothorax	0 (0%)
Identification of the pleural tattoo, *n* (%)	172 (98.3%)
Conversion to thoracotomy, *n* (%)	2 (1.1%)
Chest tube drainage time, days	1.63 ± 1.67
Postoperative length of stay, days	3.09 ± 2.14
Complications, *n* (%)	
Persistent air leak with a duration > 5 days	1 (0.6%)
Empyema	1 (0.6%)
30-day unplanned readmissions, *n* (%)	3 (1.7%)
Pathology, *n* (%)	
Malignant lesions	111 (63.4%)
Wedge resection with curative intent	104 (59.4%)
Segmentectomy	7 (4.0%)
Benign lesions	64 (36.6%)

Continuous variables are expressed as means ± standard deviations, whereas categorical data are given as counts and percentages.

**Table 3:** Summary of published studies focusing on percutaneous ICG localization of small pulmonary nodules.

Authors/Year of Publication [Reference]	Number of Patients	ICG Parameters		Tumor Characteristics		Detection Rate
		Volume, mL	Concentration, mg/mL	Mean Size, mm	Mean Depth, mm	
Ujiie et al./2017 [21]	20	0.1−0.15	0.125	12	14	90%
Zhang et al./2019 [30]	35	0.1−0.2	2.5	7	8.2	91.4%
Nagai et al./2017 [22]	37	1	12.5	9.1	9.9	95%
Anayama et al./2018 [31]	15	0.05−0.1 ^a^	0.025	10	9	100%
Rho et al./2021 [32]	24	0.3 ^b^	0.05	9 *	12 *	100%
Current study	175	0.3	2.5	8.34	5.30	98.3%

* Median value; ^a^ mixture of ICG and iopamidol; ^b^ mixture of ICG and lipiodol.

## Data Availability

Data supporting the findings from this study are available from the corresponding author upon reasonable request.
